# Special Issue “Novel Approaches to Potential COVID-19 Molecular Therapeutics”

**DOI:** 10.3390/ijms27125170

**Published:** 2026-06-07

**Authors:** Monica Gelzo, Felice Amato, Ivan Gentile, Giuseppe Castaldo

**Affiliations:** 1 Dipartimento di Medicina Molecolare e Biotecnologie Mediche, Università di Napoli Federico II, 80131 Naples, Italy; monica.gelzo@unina.it (M.G.); felice.amato@unina.it (F.A.); 2 CEINGE-Biotecnologie Avanzate Franco Salvatore, 80131 Naples, Italy; 3 Dipartimento di Medicina Clinica e Chirurgia, Università di Napoli Federico II, 80131 Naples, Italy; ivan.gentile@unina.it

Several years after its emergence, coronavirus disease 2019 (COVID-19), caused by severe acute respiratory syndrome coronavirus 2 (SARS-CoV-2) [1,2], has evolved into a persistent and complex public health issue [3]. Although widespread vaccination [4,5] and antiviral therapies [6] have significantly reduced mortality, SARS-CoV-2 continues to circulate and evolve, producing variants with altered transmissibility and immune escape potential [7]. Thus, although initially considered a viral pneumonia with occasional extrapulmonary complications, COVID-19 is now recognized as a complex disorder in which viral persistence, immune dysregulation, endothelial injury, and autoimmunity intersect in significant clinical ways [8,9]. As a result, scientific research has moved from an emergency response toward a deeper understanding of viral biology, host interactions, and innovative therapeutic strategies. The papers collected in this Special Issue contribute to a growing body of evidence showing that SARS-CoV-2 can provoke not only acute inflammation but also sustain immunological alterations that may persist well beyond the infectious phase [10,11].

A key step in SARS-CoV-2 infection is the interaction between the viral spike protein and the Angiotensin-Converting Enzyme 2 (ACE2) receptor on host cells [12]. Biskupek and Gieldon (Contribution 1) propose a two-stage recognition mechanism in which viral attachment involves a transient intermediate state between the receptor-binding domain and ACE2. Their research identified critical residues (R403, Y501, and F486) that contribute to this dynamic interaction. This model challenges the traditional view of a simple lock-and-key binding and instead describes viral entry as a dynamic multi-step process. This point of view could explain how mutations in emerging variants can modulate infectivity without necessarily increasing binding affinity. Importantly, it also suggests new therapeutic strategies targeting intermediate conformational states to eliminate the infection.

The study by Nukaga et al. (Contribution 2) introduces a new mechanism of viral-host interaction. They identified a SARS-CoV-2–derived RNA fragment capable of suppressing the human ATP5A gene via a siRNA-like mechanism leading to ATP depletion, oxidative stress, and impaired cellular maturation in cardiomyocytes, suggesting that viral RNA itself may act as a regulatory molecule within host cells. Moreover, these effects occur independently of direct viral infection, supporting the hypothesis that circulating viral RNA fragments can mediate distal organ injury such as myocardial injury [13,14].

Complementing this molecular mechanism, the study by Pius-Sadowska et al. (Contribution 3) highlights the central role of vascular dysfunction in severe COVID-19 [15]. Their findings highlight an imbalance between procoagulant and regulatory factors, particularly the vWF/ADAMTS13 axis, along with elevated VEGFR and reduced DPP-IV levels as predictors of ICU admission. These alterations suggest a state of endothelial activation, impaired angiogenesis, and nitric oxide deficiency, all of which contribute to a hypercoagulable environment. In agreement with these findings, we previously found a significant increase in serum anti-neutrophil cytoplasmic antibody (ANCA) levels in hospitalized COVID-19 patients compared to both controls and asymptomatic patients, and this increase was associated with disease severity. However, the ANCA increase was transient and none of the patients had clinical signs of ANCA-associated vasculitis except for a severe pulmonary involvement [16].

Among innovative therapeutic strategies, Asiedu et al. (Contribution 4) proposed an alternative host-targeted antiviral strategy based on mycolactone (MLN), a multitarget compound capable of interfering with SARS-CoV-2 infection [17]. They found that MLN leads to viral inhibition at nanomolar concentrations, therefore with a more effective action than antivirals such as remdesivir, and also versus multiple variants, including Alpha, Delta, and Omicron. MLN acts by disrupting clathrin-mediated endocytosis and viral fusion processes blocking both viral entry and intracellular spread. Therefore, this host-targeted approach may reduce the likelihood of resistance resulting from viral mutations.

In addition to viral entry and pharmacological interventions, the host response plays a central role in determining disease progression and clinical outcomes. Milicevic et al. (Contribution 5) describe the results obtained through transcriptomic analyses of paired nasopharyngeal swabs and blood samples collected during the acute and resolved phases of infection. Their study reveals a broad upregulation of immunity-related genes, particularly those associated with interferon signaling, along with significant alterations in hundreds of biological pathways. In agreement with these findings, we found variants in genes related to autoimmune and/or autoinflammatory diseases in about 83% of children with multisystem inflammatory syndrome, a rare severe complication of COVID-19 [18]. Ravindran et al. (Contribution 6) applied a lipidomic approach coupled with multiplex analysis to characterized lipid mediators [19] and chemokines profiles in COVID-19 patients. They found significantly increased levels of pro-inflammatory cytokines/chemokines in patients with severe disease, while anti-inflammatory lipid mediators were significantly elevated in patients with a mild phenotype. Interestingly, the increase in lipid mediators occurs earlier than cytokines, inducing anti-inflammatory mechanisms.

Finally, Yendo et al. (Contribution 7) studied the antiviral immune response in the skin, focusing on type I interferons (IFNs). Their results showed a compartmentalized immune response. In fact, IFN-β expression was increased in the epidermis, while reduced in the dermis, together with reduced expression of key signaling molecules such as STING and TLR7. This suggests that SARS-CoV-2 may differentially modulate innate immunity depending on the tissue, which could likely allow viral persistence in certain tissues. The presence of viral particles and microvascular thrombi in skin tissues further links local immune responses to systemic vascular pathology. It is noteworthy that, despite systemic hyperinflammation, skin manifestations remain relatively mild, likely reflecting a partially effective but tightly regulated interferon response. In agreement with these findings, no significant correlation was found between skin manifestations and disease severity in a single-center observational study in Italy [20].

The studies presented here collectively reinforce the idea that COVID-19 should be understood as a systemic immunovascular disorder whose consequences may extend far beyond the acute phase of infection [16,18] (Contribution 7), reminding the scientific community that SARS-CoV-2 is not simply a respiratory virus. It is an immunological stress test capable of revealing hidden vulnerabilities in host defense, vascular biology, and immune tolerance [8,21].

In conclusion, COVID-19 continues to serve as a model for modern infectious disease research. While significant progress has been made, future efforts should focus on translating molecular knowledge into effective clinical interventions, developing therapies that can resist viral evolution, and managing the long-term consequences of infection. A deeper understanding of the interaction between SARS-CoV-2 and the host will remain essential not only for managing COVID-19 but also for preparing for future emerging infectious diseases.

## Abbreviations

The following abbreviations are used in this manuscript:

COVID-19	Coronavirus disease 2019
SARS-CoV-2	Severe acute respiratory syndrome coronavirus 2
ACE2	Angiotensin-Converting Enzyme 2
ANCA	Anti-neutrophil cytoplasmic antibody
MLN	Mycolactone
